# Thyroid hormones and functional outcomes after ischemic stroke

**DOI:** 10.1186/s13044-015-0021-7

**Published:** 2015-07-04

**Authors:** Lena M. O’Keefe, Sarah E. Conway, Alexandra Czap, Carl D. Malchoff, Sharon Benashski, Gilbert Fortunato, Ilene Staff, Louise D. McCullough

**Affiliations:** Department of Neuroscience, University of Connecticut Health Center, 263 Farmington Avenue, Farmington, CT 06030 USA; Hartford Hospital, 80 Seymour Street, Hartford, CT USA

**Keywords:** Thyroid hormones, Ischemic stroke, Stroke outcomes

## Abstract

**Background:**

Stroke is the fifth leading cause of death and the primary cause of long-term adult disability in the United States. Increasing evidence suggests that low T3 levels immediately following acute ischemic stroke are associated with greater stroke severity, higher mortality rates, and poorer functional outcomes. Prognosis is also poor in critically ill hospitalized patients who have non-thyroidal illness syndrome (NTIS), where T3 levels are low, but TSH is normal. However, data regarding the association between TSH levels and functional outcomes are contradictory. Thus, this study investigated the role of TSH on stroke outcomes, concomitantly with T3 and T4.

**Findings:**

In this work, blood was collected from patients with radiologically confirmed acute ischemic stroke at 24±6 hours post-symptom onset and serum levels of TSH, free T3, and free T4 were measured. Stroke outcomes were measured at discharge, 3 and 12 months using the modified Rankin scale and modified Barthel Index as markers of disability. Though we found that lower levels of free T3 were associated with worse prognosis at hospital discharge, and at 3 and 12 months post-stroke, none of these outcomes held after multivariate analysis. Thus, it is likely that thyroid hormones are associated with other factors that impact stroke outcomes, such as sex, age and stroke etiology.

**Conclusions:**

This study found that lower levels of free T3 were associated with poorer outcomes at hospital discharge, and at 3 and 12 months post stroke, however, these associations diminished after correction for other known predictors of stroke outcome. Thyroid hormones have a complex relationship with ischemic stroke and stroke recovery, which merits further larger investigations.

## Introduction

Stroke is the fifth leading cause of death and the primary cause of long-term adult disability in the United States [1]. Perturbations in the hypothalamus-pituitary-thyroid (HPT) axis affect stroke risk and stroke outcomes. Hypothyroidism can cause hypertension, hypercholesterolemia, cardiac dysfunction, and both hypo- and hypercoagulability, all of which are risk factors for stroke [2–4]. Hyperthyroidism is also associated with atrial fibrillation, which is a common cause of cardioembolic stroke [5].

The relationship between thyroid hormones and functional outcomes post-stroke is complex. Current data has shown that low T3 levels immediately following acute ischemic stroke (AIS) are associated with greater stroke severity and mortality, and poorer functional outcomes [6–8]. This is also true in critically ill hospitalized patients who have non-thyroidal illness syndrome (NTIS; or ‘euthyroid sick syndrome’), where T3 levels are low, but TSH is normal [9–11]. NTIS patients have poorer short-term prognosis and higher mortality rates at 12 months compared to non-NTIS patients [6]. However, data regarding the association between TSH levels and functional outcomes after stroke are conflicting [12–15]. The association between TSH levels and stroke outcome remains unclear, in part, because few studies have looked at TSH, T3, and T4 values concurrently. Doing so may be especially relevant in patients with brain injury and perturbations in HPT signaling.

This study examines the association between thyroid hormone levels (T3, T4, and TSH) and ischemic stroke outcomes as determined by the modified Rankin scale (mRS) and modified Barthel index (mBI) at hospital discharge, and at 3 and 12 months.

## Methods

### Study setting and population

This study was conducted at an 868-bed community-based teaching hospital with a Neurology Residency Program. This center has The Joint Commission’s Gold Seal of Approval and the American Heart Association’s Advanced certification as a comprehensive stroke center and has an annual stroke admission rate of approximately 1000. This study was approved by the Institutional Review Board.

### Study protocol

Blood was collected from patients over 18 years of age with radiologically confirmed acute ischemic stroke (AIS) (*n* = 129) at 24 ± 6 h post-symptom onset under an approved IRB from December 2011 to May 2013. Patients with active malignancy or treatment with immunosuppressants were excluded from the study. Serum levels of TSH, free T3 (fT3), and free T4 (fT4) were quantified by solid-phase chemiluminescent immunometric assay (R&D Systems, Minneapolis, MN).

### Measurements

Trained nursing staff prospectively collected clinical and patient information and entered it into the Stroke Center’s database, which contains information regarding patient presentation, stroke etiology, and outcome. Baseline demographic information (age, sex, medical history, medication use) was collected. Risk factors such as history of hypertension, atrial fibrillation, coronary artery disease, previous stroke or transient ischemic attack, diabetes mellitus, hypercholesterolemia, cigarette smoking and alcohol use were also collected. Primary outcomes were in-hospital mortality and National Institutes of Health Stroke Scale (NIHSS) score on admission. Secondary outcomes included death or hospice after discharge, and modified Rankin scale (mRS) and modified Barthel Index (mBI), at 3 and 12 months post-stroke. The mRS is a scale for measuring the disability of patients post-stroke. It has a scale of 0–6, with 0 designating no limitations or symptoms, 5 being severe disability requiring constant care, and 6 being dead [16]. The mBI is a scale used to measure performance in activities of daily living. It is scored on a scale of 0–20 with 20 being independent and 0 being completely dependent [17].

### Statistical analysis

Continuous data, such as age, are presented as means (standard deviation). Non-normally distributed continuous and ordinal data are presented as medians (interquartile range) and were analyzed with non-parametric tests (Spearman’s Rho). Categorical data are presented as proportions, and group differences were assessed with chi-square tests of proportion. Several variables with non-normal distributions were dichotomized and analyzed with chi-square test of proportions. These included the mBI, dichotomized into “independent” (score of 15 or greater), or “dependent” (14 or less), and mRS, dichotomized into “disabled” (score of 3 or greater) or no disability to slight disability, termed “abled” (2 or less). A “poor outcome” composite measure was defined as death or disabled with an mBI ≤14 or a mRS ≥3 as defined above at 3 and 12 months after AIS. A “favorable outcome” composite measure was defined as mBI ≥15 or mRS ≤ 2. Significant findings identified in the univariate analyses that pertained to more than one thyroid marker were reanalyzed using multivariable logistic regression to control for all significant confounding variables. Thus, a unified list of predictive factors was applied to all three thyroid markers in multivariate analysis. This included atrial fibrillation (AFIB), depression, urinary tract infection (UTI), intra-arterial devices (IA), age and NIHSS on admission. The criterion of statistical significance was set at 0.05. All analyses were performed using Statistical Package for the Social Science v14.

## Results

### Demographics

Patient demographics are listed in Table 1. Out of the 129 patients, 119 were classified as euthyroid (TSH between 0.27 and 4.20 microunits/L), 7 as hyperthyroid (TSH < 0.27 microunits/L), and 3 as hypothyroid (TSH > 4.20 microunits/L).

**Table 1:** Characteristics of study patients

Category	
Age (y ± sd)	67.03 ± 14.474
Sex	
Female	51 (39.5 %)
Male	78 (60.5 %)
Ethnicity	
White	99 (76.7 %)
Black	12 (9.3 %)
Latino	15 (11.6 %)
Other	3 (2.3 %)
History of	
HTN	106 (82.2 %)
Diabetes	46 (35.7 %)
Heart disease	44 (34.1 %)
Stroke	19 (14.7 %)
High cholesterol	91 (70.5 %)
Atrial fibrillation	31 (24 %)
Angina	0
Blood clots	4 (3.1 %)
Arthritis	16 (12.4 %)
Seizures	5 (3.9 %)
Dementia	4 (3.1 %)
Depression	17 (13.2 %)
Lung problems	8 (6.2 %)
Aspiration pneumonia	9 (7.0 %)
Aneurysm	0
UTI	20 (15.5 %)
Smoking	25 (19.4 %)
Alcohol	5 (3.9 %)
Drugs	2 (1.6 %)
Origin Location	
Home	61 (4.3 %)
Community	16 (12.4 %)
Inpatient	1 (0.8 %)
Transfer patient	44 (34.1 %)
Facility	6 (4.7 %)

Characteristics of patients enrolled in this study including demographics, past medical history, and origin location

*HTN* hypertension, *UTI* urinary tract infection

### Demographic associations

As expected TSH was inversely correlated with fT4, though there was no significant correlation between TSH and fT3. fT3 and age were inversely correlated, but there was no significant association with age for TSH or fT4. There was no significant difference between males and females in TSH, fT4, or fT3 levels (Table 2).

**Table 2:** Associations between thyroid hormones and patient demographics, baseline function, stroke severity, medical history, and medication usage

	TSH	fT3	fT4
Demographic associations			
TSH	NS	NS	*R* = −0.246*
Sex	NS	NS	NS
Age	NS	*R* = −.332***	NS
Pre-stroke associations			
Baseline mRS	NS	*R* = −0.393***	NS
Baseline mBI	NS	*R* = 0.295***	NS
Admission associations			
Admission NIH	NS	*R* = −0.191*	NS
Hospital mRS	*R* = −0.206	NS	*R* = 0.176*
Admission mBI	NS	NS	NS
Other associations			
Past medical history			
Hypertension	NS	NS	NS
Heart disease	NS	NS	NS
Prior stroke	NS	NS	NS
Diabetes	NS	NS	NS
Depression	NS	NS	NS
Alcohol use	NS	NS	NS
Medications			
Anti-platelets	NS	NS	NS
Anticoagulants	NS	NS	NS
Anti-hypertensives	NS	NS	NS
Anti-cholesterol	NS	NS	NS

Associations between thyroid hormones and demographics upon hospital admission. Patients with low levels of fT3 had increased disability and poorer pre-stroke function. NIH score on admission was inversely associated with fT3. Hospital mRS was inversely associated with TSH and directly associated with fT4. None of the thyroid hormones were associated with past medical history of hypertension, heart disease, prior stroke, diabetes, depression, alcohol use, or with the usage of anti-platelets, anti-coagulants, anti-hypertensives, or anti-cholesterol medications

*p < 0.05; **p < 0.01; ***p < 0.001

### Pre-stroke associations

Patients with low levels of fT3 had increased disability at baseline indicated by higher pre-stroke mRS scores (*r* = −0.393, *p < 0.001*) and lower mBI scores (*r* = 0.295*, p = 0.002*) (Table 2).

### Admission

The NIHSS on admission was inversely associated with fT3 (*R* = −.191, *p = 0.031*), but not TSH or fT4. Hospital mRS was inversely associated with TSH (*R* = −0.206, *p = 0.019*), and directly associated with fT4 (*R* = 0.176, *p = 0.045*). There was no association with fT3. Admission mBI score was unrelated to fT3, fT4, or TSH levels (Table 2).

### Outcome at discharge

Patients with lower TSH had significantly higher in hospital mortality (*p = 0.015*). There was no association between hospital mortality and fT3 or fT4. However, patients who were alive at discharge had significantly higher fT3 levels than patients who either died in hospital or went to hospice (*p = 0.01*) (Table 3).

**Table 3:** Outcomes

	TSH	fT3	fT4
Discharge outcomes			
Death in hospital		NS	NS
Died	0.5245 (0.303–0.761)*		
Alive	1.275 (0.75–1.9875)*		
Death in hospital or hospice vs alive	NS		NS
Death or hospice		1.99 (1.74–2.475)*	
Alive		2.47 (2.13–2.95)*	
3 month outcomes			
Death vs alive at 3 months		NS	NS
Death	0.66 (0.372–1.45)*		
Alive	1.27 (0.75–2.11)*		
Independence level	NS		NS
mBI > 15, independent		2.53 (2.24–2.99)**	
mBI < 14, dependent		2.06 (1.85–2.6)**	
Poor outcome, mRS > 3 or death	0.873 (0.586–1.48)*		
Favourable outcome, mRS < 2	1.54 (0.6575–2.255)*		
12 month outcomes			
mRS score	NS	*R* = −0.268*	NS
mBI > 15, independent		2.525 (2.26–2.965)*	
mBI < 14, dependent		2.175 (1.865–2.823)*	
Poor outcome, mRS > 3 or death		2.04 (1.825–2.485)***	
Favourable outcome, mRS < 2		2.62 (2.32–2.97)***	

Patients with better acute and long term outcomes had higher TSH levels, and higher fT3 levels at 3 and 12 months post-stroke. No associations were seen between acute and long term outcomes with fT4

*p < 0.05; **p < 0.01; ***p < 0.001

### 3 month outcomes

Low TSH was related to 3 month mortality (*p = 0.036*). Patients who died at 3 months had lower TSH values than those who were alive at 3 months. Higher 24 h fT3 levels were predictive of good outcome at 3 months as measured by mBI > 15 *(p = 0.004)*. In addition, patients with a favorable composite outcome at 3 months had higher TSH levels than those with a poor composite outcome (Table 3). There was no association between outcome and fT4 (Table 3).

### 12 month outcomes

The same pattern was seen at 12 months. Patients with the highest fT3 levels were the most likely to be independent as measured by the mBI 1 year after ischemic stroke (*p = 0.042*). This was confirmed by an inverse association between mRS and fT3 levels, showing that the higher the initial fT3, the better the outcome, even a year after the index event (*p = 0.042*). Neither mBI nor the mRS at 12 months was related to TSH or fT4 levels (Table 3).

### Other associations

There were differences in fT3 based on stroke etiology determined with the TOAST classification. Patients with small vessel disease had higher fT3 values than those with large vessel or cardioembolic stroke etiology (H(2) = 11.06, *p = 0.004*) with a mean rank of 83 for small vessel disease, 59 for large vessel disease, and 53 for cardioembolic stroke etiology.

### Multivariate

A multivariate logistic regression model was used to control for the potential confounders identified in the univariate analyses as described above. When controlling for AFIB, depression, UTI, IA, age and NIH on admission, thyroid markers were not associated with mortality or functional outcomes (Table 4), suggesting that thyroid status is related to other risk factors rather than an independent predictor of outcome.

**Table 4:** Multivariate analysis

	TSH: OR (95 % CI), P	fT3: OR (95 % CI), P	fT4: OR (95 % CI), P
Death in hospital or hospice	0.635 (0.250–1.613), NS	1.06 (0.569–1.974), NS	1.957 (0.323–11.872), NS
3 month outcome, mRS > 3 or death vs mRS < 2	0.686 (0.398–1.181), NS	0.517 (0.226–1.185), NS	1.574 (0.465–5.328), NS
3 month outcome, mBI > 15 vs mBI < 14 or death	0.707 (0.443–1.127), NS	0.513 (.215–1.225), NS	

Multivariate regression results between thyroid hormones and acute and long-term outcomes post-stroke. When controlling for AFIB, depression, UTI, IA, age, and NIH on admission, thyroid markers were not associated with mortality or functional outcomes

## Discussion

This study is the first to look at TSH, fT3, and fT4 concurrently and their association with stroke severity and long term functional outcomes. This study found that low TSH and fT3 were associated with poor function at 3 months, and that low TSH and fT3 were associated with higher rates of death in the hospital. However, these results were not seen in the multivariate analysis when other known predictors of outcome such as stroke severity (NIHSS) were controlled. This is likely due to the relatively small sample size of the study, and the fact that the majority of patients sampled were euthyroid. Though prior studies have demonstrated an association between low T3 levels and poor outcomes, it is likely that this study was underpowered to detect an association. Outcomes at 12 months were only available in a minority of patients. Future studies with an increased number of patients with hyper- and hypothyroidism and hypothyroidism with hormone replacement should be done to assess a wider range of thyroid values and outcomes. In addition, assessment of hormone levels over multiple time points is needed to demonstrate changes in thyroid status over time, as only one time-point was evaluated here. However, the significant association between thyroid hormone levels and outcome in the univariate models suggest that this is worth pursuing vigorously in larger studies, as strategies to replace thyroid levels could be developed for therapeutic trials.

Although it is unknown whether administration of exogenous thyroid hormone in stroke patients improves outcomes, administration of T3 resulted in increased mRNA expression of reelin and brain-derived neurotrophic factor in rats, both of which are important in nervous system regeneration [18]. In hospital patient monitoring of thyroid status may be important as higher levels of fT3 have been associated with better functional outcomes. Therefore, treatment to normalize and/or elevate fT3 values for patients presenting with NTIS could be beneficial to a patient’s recovery after acute ischemic stroke.

## Abbreviations

HPT: Hypothalamus-pituitary-thyroid axis
AIS: Acute ischemic stroke
NTIS: Non-thyroidal illness syndrome
mRS: Modified Rankin scale
mBI: Modified Barthel index
NIHSS: National Institutes of Health Stroke Scale
AFIB: Atrial fibrillation
UTI: Urinary tract infection
IA: Intra-arterial devices
fT3: Free T3
fT4: Free T4
